# How Professional and Market Logics and the Conflict between Institutional Demands Affect Hospital Accreditation Compliance: A Multiple-Case Study in Brazil

**DOI:** 10.3390/healthcare12090914

**Published:** 2024-04-27

**Authors:** Tiago Martins Ramos da Silva, Luciano Rossoni

**Affiliations:** 1 Graduate Program in Administration, University of Grande Rio, Duque de Caxias 25071-202, Brazil; tiago.ramos@fiocruz.br; 2 Oswaldo Cruz Foundation, Fernandes Figueira Institute, Rio de Janeiro 22250-020, Brazil; 3 Graduate Program in Administration, University of Brasilia, Brasilia 70910-900, Brazil

**Keywords:** hospital accreditation, institutional logics, organizational practices, strategic responses, institutional theory

## Abstract

Hospital accreditation has become ubiquitous in developing countries. While research acknowledges that accreditation can enhance healthcare quality, efficiency, and safety, concerns persist regarding hospitals’ management of conflicts stemming from the diverse institutional logic inherent in this process. Therefore, this study aimed to investigate how professional and market logic, alongside conflicts arising from institutional demands, affect compliance with hospital accreditation. To this end, we conducted a multiple-case study in four Brazilian hospitals employing in-depth interviews and on-site observations. The triangulation of narrative analysis and the outcomes of multiple correspondence analysis revealed that when professional logic predominates, there is a greater propensity to tailor accredited activities by segmenting the tasks between physicians and nurses with the intention of mitigating existing conflicts. Conversely, when conflicts occur over established goals between professionals and orientated marked logic executives, the accreditation process is impeded, resulting in non-compliance. Ultimately, the findings underscore the alignment between the pursuit of legitimacy and efficiency within the accreditation process. We conclude by delineating the theoretical and practical implications of scrutinizing the internal dynamics of institutional logic.

## 1. Introduction

Hospital organizations worldwide, including those operating in emerging countries, have faced mounting pressure to adhere to healthcare standards endorsed by hospital accreditation programs [1,2,3,4,5]. Such programs entail an evaluation process conducted by a certifying body to ascertain whether hospitals comply with rigorous quality and safety standards in healthcare delivery, thus attesting to their efficacy [6,7]. However, despite the acknowledgment that such programs drive relevant structural changes within hospitals, doubts persist regarding their effectiveness [2,5,8,9].

Studies indicate that the effectiveness of hospital accreditation hinges on factors such as management commitment, resource accessibility, team leadership, and integration between professionals [5,10] that impede these processes [2]. Seeking to fill this gap, this article focuses on the intricate dynamics surrounding hospital accreditation, exploring the conflicting influences of market and professional logic that manifest in the day-to-day operations of hospitals. To this end, we draw upon the literature on institutional logic within organizational theories [11,12], which posits that the process of change caused by the adoption of accreditation practices engenders a dual focus. On the one hand, market logic predominates, emphasizing economic and business concerns, with a primary emphasis on objectives and efficiency metrics [13,14,15]. On the other hand, a professional logic perspective underscores aspects related to the practice and identity of healthcare professionals [2,16], who are immensely concerned with the processes involved in patient care [1,17]. Against this backdrop, this study aims to explore how professional and market logic, alongside the conflicts arising from institutional demands, impact compliance with hospital accreditation.

Recognizing the inherent contradictions between market and professional logic in accreditation compliance and necessitating a complex research approach [13], we developed a qualitative multiple-case study encompassing four Brazilian hospitals, where we interviewed certification agency auditors, managers, physicians, nurses, and other professionals. Initially, we explored the relationship between the prevailing logic and objectives established for adoption, which revolves around the pursuit of efficiency and legitimacy within the domain [18,19]. Subsequently, we examined hospitals’ conformity, customization, and non-conformity responses to accreditation norms and standards [19,20,21,22]. Thirdly, we identified the conflicts arising at both the means level—accentuating tensions between hospital care practices and certified protocols—and the goals level, where conflicts emerge between senior management’s financial objectives and the resource requirements for certification fulfillment [14,23]. Lastly, acknowledging the duality between the material and symbolic dimensions of hospital accreditation, we scrutinized the interplay among various analytical categories—logic, adoption objectives, strategic responses, and nature of demands—through a triangulation approach combining narrative analysis [24] and multiple correspondence analysis (MCA), as such aspects intricately shape the practical implementation of accreditation [25].

We contend that managers and professionals aligned with market and professional logic tend to espouse distinct perspectives on the implications of accreditation, both in terms of how to implement these strategies (means) and their underlying rationales (goals) [23]. As such, these logics may prove incompatible, prompting their segmentation across different sectors within hospitals to mitigate extensive conflict [26,27]. Notably, in scenarios where professional logic prevails, conflicts are expected to arise primarily at the mean activities, fostering a drive towards customization. Conversely, under the dominance of market logic, conflicts typically surface at the goals level, resulting in non-conformity. Indeed, this study aimed to elucidate how professional and market logic, alongside conflicts stemming from institutional demands, impact hospital accreditation compliance.

This study offers significant contributions to the existing literature on hospital accreditation and institutional logic by analyzing the facets raised in this argument. Firstly, while prior research has primarily focused on evaluating compliance in broad strokes [18], our study advances by delving into the intricate interplay between market and professional logic within the specific realm of hospital accreditation [23,27]. Secondly, departing from studies that oversimplify compliance as a binary issue of compliance or noncompliance, our inquiry aims to unravel how practices become contested by different actors whose logic inherently clash [26]. By doing so, we offer a deeper understanding of how hospital organizations grapple with compliance and the challenges of customizing practices within an environment where multiple logics often intersect [2,14]. Thirdly, by emphasizing the distinction between demands on means and goals, our study bridges a gap in the literature, shedding light on how specific demands affect accreditation [23]. Lastly, by examining how hybrid logics manifest in the context of accreditation, we demonstrate that conflicts arising from divergent approaches to the means by which activities are carried out often lead to segmentation [27] of practices between physicians and nurses. Indeed, when conflicts occur over goals, they tend to impede accreditation efforts driven by market logic [23,28].

We have structured this article into five sections in addition to the introduction. In the first segment, we present the foundations of institutional logic, pointing out that hospital accreditation practices are shaped by two competing logic—market and professional—whose nature of demands, strategic responses, and adoption objectives may vary according to the emergency of such logic. Subsequently, we elaborate on the methodological aspects, highlighting the analytical categories and the triangulation between methodologies. Next, we present the findings of narrative analysis and multiple correspondence analysis, which are then synthesized into a set of empirically verifiable propositions in the discussion section. Finally, we delineate the theoretical and practical implications, highlighting limitations and offering suggestions for future research, culminating in a conclusion.

## 2. Theoretical Framework

### 2.1. Isomorphism, Dissimilarity, and Institutional Logics

One of the paramount inquiries in organizational institutionalism revolves around understanding why organizations tend do become increasingly homogeneous [29]. A key aspect of this phenomenon lies in the need for a social mandate wherein organizations conform to standards deemed legitimate [30], such as hospital accreditation, for example. As a consequence, the proliferation of such norms drives organizations towards greater isomorphism.

In contrast to this assumption, this study endeavors to elucidate the isomorphic process within Brazilian accredited hospitals and explore the variances in the adoption of such practices. We contend that hospital accreditation remains a contested practice [30] among two factions of stakeholders who espouse different logics regarding the significance of such practices: on the one hand, physicians, nurses, and healthcare professionals advocate a professional logic, while on the other, executives and managers champion a market logic [28]. Hence, contestation of the accreditation process by stakeholders aligned with different logic can engender customization and non-conformity rather than mere compliance.

Institutional logic represents “an order of production, composed of distinct individuals and objects mediated by a regime of material practice. Institutional logics point to […] orders of practice that depend on the identities of individuals and ontologies of objects, which, in turn, depend on these same orders of practice” (p. 336, [25]). This definition underscores the material and symbolic dimensions inherent in the adoption of organizational practices, including hospital accreditation [13,14], which, in turn, are shaped by the identity and interests of members engaged in the accreditation process [2,16].

According to Thornton et al. [12], the idea of institutional logic was introduced into organizational studies by Alford and Friedland [31] and further elaborated by Friedland and Alford [11] to understand the diverse practices and beliefs shaping institutions in Western society. Friedland et al. [25] argued that institutional logics are underpinned by institutional substances (or objects). These substances are not tangible or material but rather represented by unobservable rationales that can only be apprehended through practice. Therefore, practice emerges as the explicit manifestation of these institutional substances.

In addition to portraying everyday practice as encompassing symbolic elements such as values and interests, the institutional logic approach highlights both the contradictions between different perspectives of groups within organizations [32] and the array of demands that organizations must adapt to [33]. Consequently, the presence of multiple logics amplifies institutional complexity [27], especially during organizational change processes, such as hospital accreditation [17].

This study underscores the conflicts arising from divergent logic when they manifest competing aspects. This occurs because resistance to institutional change arises from both the interplay between logic as well as the amalgamation of environmental pressures [34], the institutional clout of influential actors [10,13], and the strategic objectives of the organization [16,23].

In the hospital context, research suggests that competing institutional logics explain how hospital organizations respond to the regulatory process [1] and can influence the strategies and practices they adopt [33]. Furthermore, regarding hospital accreditation, prior studies indicate that the clash between institutional logic can heighten resistance to adoption, as physicians and nurses strive to maintain their identity while resisting market pressures [2,13,15].

While distinct institutional logics exist, their coexistence does not inevitably lead to competition, as the convergence or even the formation of hybrid forms can occur, especially under the influence of pressures to adopt practices endorsed by external agencies, such as hospital accreditation [2,14].

### 2.2. Hospital Accreditation as an Institutional Standard

Organizational institutionalism offers a rich research agenda for analyzing the adoption of organizational practices [18,21], mainly when such practices pertain to certification processes [19]. Numerous studies have delved into the adoption of quality certifications, such as ISO9000, ISO14001, ISO31000, and various iterations of total quality management [35,36], as well as environmental and sustainability certifications [37].

This body of literature asserts that the certification process, beyond its aim to improve quality and productivity, which relates to efficiency considerations, also serves as a signal that the organization meets socially mandated criteria [38]. Indeed, hospital accreditation processes follow a similar trajectory [2,22].

Within the healthcare literature, there is widespread discussion surrounding the institutional dimensions of accreditation, often centering on the pursuit of quality in hospital care [3,39,40], tracing its origins from the early 20th century in the United States to its global prevalence today [4,41]. In Brazil, the first endeavor focused on assessing quality in hospital organizations was in 1935, when Odair Pedroso developed a form to evaluate hospital quality for the now-defunct Hospital Assistance Commission of the Ministry of Health [42].

Since then, several initiatives have been undertaken, albeit progress has been gradual. It was only in 1989 when the World Health Organization (WHO) emphasized the importance of accreditation for enhancing healthcare quality in Latin America, that this process gained momentum in Brazil. Almost a decade later, in 1998, the Brazilian Accreditation Commission (CBA) was established as the Brazilian representative of the Joint Commission International. A year later, the National Accreditation Organization (ONA) was founded, primarily as a domestic entity, following the release of the Brazilian Accreditation Manual. While other accrediting bodies have emerged since then, ONA and CBA stand out in the country as pioneers with a large clientele.

ONA defines hospital accreditation as a system for evaluating and certifying the quality of health services, characterized by its educational nature, voluntary participation, and absence of supervisory or regulatory purposes. It is pertinent to note that both ONA and CBA are private organizations, and their adherence to the accreditation process is voluntary and requires significant financial investment. This definition of hospital accreditation aligns with the prevailing literature, which views it as an evaluation of structures and processes conducted by an independent accreditation body to ascertain whether a hospital meets the requisite quality and safety standards [5].

The accreditation process engages the entire hospital team, including physicians, nurses, technicians, directors, managers, etc. Consequently, accreditation agencies adhere to a stringent manual containing a series of protocols essential for hospital accreditation. This process stands apart from other certifications due to its exclusivity to the health sector, entailing more robust standards of ethics and morality [5], along with a greater emphasis on technical proficiency and effectiveness.

While the pursuit of quality is overtly stated as one of the primary objectives of the accreditation process [40], hospital organizations undoubtedly harbor other objectives that may influence the results. In this vein, the extent to which institutional logic shapes and even formulates these objectives is a key question this study seeks to address. However, what exactly is the relationship between institutional logic and the outcomes achieved and anticipated through the hospital accreditation process?

### 2.3. Institutional Logics Constituting the Hospital Accreditation Process

Previous studies leveraging organizational institutionalism to scrutinize the adoption of quality management practices underscored the pursuit of efficiency and legitimation [36,43]. Moreover, in the past decade, studies have delved into understanding how various social orders in society—such as the capitalist market, the bureaucratic State, families, democracy, and religion, each characterized by a distinct logic—can legitimize hospital activities [16,17].

Such studies on institutional logics within the healthcare domain, alongside seminal studies, has identified two prevailing logics in healthcare organizations: market logic, interested in the commercial exploitation of hospital activity, and professional logic, which pertains to aspects associated with the delivery of medical care within hospitals [35,44,45]. The essence of these logics is delineated in Table 1.

The specific technical expertise of healthcare professionals reinforces professional logic, while simultaneously, the imperative for a market-oriented approach to hospital management is becoming increasingly pronounced in healthcare organizations [1]. Given these divergent orientations, uncertainties persist regarding how these logics function in hospital accreditation compliance. In the pursuit of quality certifications, a prevalent endeavor in the hospital realm, how do these logics help elucidate organizational adoption decisions?

Drawing upon the concepts elucidated by Friedland et al. [25], professional logic can be construed as an institution comprised of a substance (the profession itself), forms of subjectivity (emotions such as protection, dedication, zeal, and competition), and a set of practices (behaviors such as exercising caution at work, pursuit of quality, and defense of professional rights). Aspects such as relational networks, professional associations, status, and the quest for quality [12] are prevalent and evident traits within healthcare organizations. Therefore, individuals possessing technical expertise wield significant influence over organizational decision-making processes.

Conversely, under market logic, private hospital organizations pursue profitability and, correspondingly, endeavor to minimize costs, increase efficiency and competitiveness, and reach more customers. Indeed, market logic manifests as an institution characterized by a substance (the market itself), forms of subjectivity (traits such as competitiveness, opportunism, and self-interest), and a repertoire of practices (cost reduction measures, investments, increased production, and emulation). In the hospital context, various logics coexist, giving rise to divergent responses to hospital accreditation due to inherent contradictions among them.

### 2.4. How Do Hospitals Respond to the Hospital Accreditation Process?

The question of why organizations adopt certain practices remains a persistent inquiry in organizational studies. Apart from technical and efficiency-related concerns, organizations adopt practices to legitimize themselves [38,46]. Thus, when organizations implement new practices such as hospital accreditation, their aim is to attain legitimacy within their respective audiences: “a generalized perception or assumption that an entity’s actions are desirable or appropriate within a structure of norms, values, beliefs, and socially constructed beliefs” (p. 574, [47]).

Despite the significance of legitimacy for organizations, many practices deemed legitimate are adopted without effectively addressing actual organizational demands, resulting in ceremonial exercises. For example, displaying a certificate on the wall may confer prestige and acceptability, yet managers may not even discern its practical benefits for organizational activities. It is important to note, however, that we are not discrediting the necessity of legitimacy here, as something deemed legitimate does not necessarily need to be ceremonial [48].

From the perspective of potential ceremonial responses, Oliver [21] developed a typology of strategic responses to institutional pressures, which has influenced investigations in the hospital domain [18,19,20]. These responses vary in terms of conformity and resistance and are categorized as acquiescence, compromise, avoidance, defiance, and manipulation.

While Oliver’s [21] theoretical framework is valuable, it has limitations, particularly in the context of Brazilian hospital accreditation, which is voluntary. This implies that hospitals face minimal external regulatory pressure from the State or other oversight organizations, thereby amplifying the visibility of normative and cognitive pressures from professionals and clients. Nevertheless, despite the absence of obligation and limited customer awareness about this process, internal pressures remain predominant and robust.

Voluntarily, the hospital organization must adhere to the protocols and practices pre-established by the certifying organization until accreditation is obtained. However, after this initial period, there is no assurance that these protocols will continue to be followed, potentially leading to the adoption of camouflage practices or ceremonial gestures when interacting with external actors, except when the renewal of certification is necessary. Additionally, accreditation does not only serve to confer benefits such as eligibility for credit programs and participation in associations; it also facilitates various aspects of hospital operations. According to ONA [49], hospital accreditation “has an eminently educational character, aimed at continuous improvement, without the purpose of inspection or official/governmental control, and should not be confused with licensing procedures and typical actions of State (p. 12)”.

These characteristics of accreditation diverge, in part, from the elements conceptualized in Oliver’s study [21], which does not imply, however, that the sole response to such certifications is mere acceptance. That is why we have simplified organizational responses, aiming to render the analysis more objective and coherent with the research object: *Conformity* when the hospital fully complies with the accreditation process; *Non-conformity*, when the hospital fails to meet the minimum requirements of the process; and *Customization* when the hospital partially fulfills the needs of the accreditation process, but tailors certain actions, according to its convenience.

In addition to examining strategic responses, understanding the demands imposed by accreditation in its adoption is imperative. Thus, we consider the nature of these demands: whether they pertain to the means of activities or whether they relate to the desired goals [23].

Identifying the nature of these demands enables us to pinpoint areas of conflict, whether they arise in the execution of the means to achieve the goals set by the organization or in determining which goals the organization should pursue [23]. *Conflict over means* entails clashes or disagreements over the methods employed to achieve a goal. In contrast, *Conflict over goals* involves disputes or contradictions regarding which outcomes are deemed legitimate to pursue [23].

Both the model proposed by Oliver [21] and the framework developed by Pache and Santos [23] contribute significantly to identifying possible responses or behaviors of organizations in the face of competing logic. However, these models do not assess the significance or influence of specific logics on organizational responses, thus hindering the ability to discern whether different logics may elicit different responses.

For this reason, we analytically contend that organizational responses to the practice of hospital accreditation (conformity, customization, and non-conformity) are contingent upon organizational objectives (efficiency and legitimacy), prevailing competing logics (professional and market), and the nature of demands (in the means or the goals). It is crucial to acknowledge the potential competition of institutional logics within hospital organizations during the accreditation process, as different institutional logics coexist [12]. 

Therefore, this study underscores the predominant logic within the hospital environment [13,44], ensuring that the analytical depiction of logic offers a more robust framework for analyzing the outcomes. Additionally, we modeled the study under the premise that logics must be examined as practical achievements, where material demands exhibit a duality with symbolic issues [50,51], prompting us to adopt a triangulation approach combining qualitative data with formal relational methods of analysis [25].

## 3. Materials and Methods

As the primary objective of this research was to elucidate the mechanisms through which institutional logic and the nature of demands affect hospital accreditation in terms of alignment with objectives and types of strategic responses, we opted for the multiple-case study strategy [52,53]. This selection stemmed from the necessity to understand the intricacies of such processes through an inductive approach involving categorization and analysis of interviews and observations conducted at the research site [54].

Furthermore, as the literature had already delineated the dominant logic regarding the hospital accreditation process [1,2,13,14], along with other relevant analytical categories for analysis, we adhered to the methodological guidelines for capturing institutional logic by Reay and Jones [55]. These authors stipulate that qualitative material should be analyzed on pre-established analytical categories in such circumstances based on previously defined analytical categories. Despite commencing with a set of a priori categories, we applied inductive logic to formulate propositions based on the analysis of qualitative data obtained from interviews and observations [56], as explained in Section 3.4. Subsequently, we compared the qualitative narratives with the outcomes of the multiple correspondence analysis outlined in Section 3.5 to account for the complex and relational nature of institutional change processes driven by diverse logics [37].

### 3.1. Selection of Hospitals, Participants, and Field Research

Initially, we conducted a preliminary immersion in various accredited hospitals that had recently completed the accreditation process to negotiate access to the target organizations under investigation [57]. This step allowed us to discern the differences in the accreditation processes across hospitals. In Rio de Janeiro, Brazil, there are 35 hospitals accredited by the National Accreditation Organization (ONA, see https://ona.org.br/, accessed on 15 April 2024) and eight (8) accredited by the Brazilian Accreditation Consortium (CBA, https://cbacred.org.br/, accessed on 15 April 2024). After selecting the hospitals most likely to provide valuable insights, we negotiated access to the field with their managers. This process was facilitated by one of the researchers, who has extensive experience in health management spanning over a decade.

Consequently, we selected four hospitals in Rio de Janeiro, Brazil: two accredited by ONA and two by the CBA. The rationale behind selecting these two accrediting organizations lies in their established presence in the field and their substantial number of accredited hospitals in Brazil. When selecting hospital case studies, we focused on criteria such as representativeness and relevance to research questions [58]. Factors including accreditation issues, innovative care practices, and administrative challenges were considered. Therefore, we chose hospitals that could offer detailed insights and clearly illustrate pertinent processes and outcomes. We assessed the representativeness of each case, the accessibility of data, uniqueness, and the potential for generalizing findings.

Before commencing the field research, we extracted information about the accreditation process from the ONA and CBA manuals, including evaluation forms and the minimum requirements for obtaining certification. Next, we conducted in-depth interviews with certification agency auditors from each accrediting organization to enrich our understanding of the accreditation process and to address any uncertainties arising from the manual readings.

Table 2 outlines the subjects involved in the field research, comprising managers directly engaged with the accreditation process, who posed 16 guiding questions. Additionally, physicians, nurses, and physiotherapists involved in the daily implementation of accreditation in their respective hospitals openly answered 11 questions (Table 3). The study enlisted 18 participants, including 16 hospital staff and executives and two auditors from each certifying body. The interview questionnaire was specifically tailored for this study. Multiple interviews were conducted with each respondent to complement information and triangulate evidence during the qualitative observation process. 

During the field research, apart from conducting interviews, we had the opportunity to observe the daily operations of the hospitals and witness the evaluation processes carried out by the accrediting organizations.

### 3.2. Ethical Considerations

The study did not need to require approval from the research ethics committee of the University of Grande Rio, as per the university’s regulations, which do not mandate committee review for studies posing no risks for participants, provided that the researchers comply with the guidelines outlined in the CNS/MS Resolution No. 466/2012.

Both hospitals and respondents provided an informed consent form by signing a research consent form. This form outlined the objective of the research, its strictly academic nature, and the assurance of participants’ rights to confidentiality and withdrawal. Participant anonymity was maintained by using professional titles and assigning identifying numbers, with hospitals designated by letters (e.g., Physician 1 at Hospital B).

Finally, it should be noted that the researchers have no conflicts of interest, as they are not directly affiliated with the accredited hospitals or the accreditation agencies. Furthermore, they do not stand to benefit directly or indirectly from the study’s results.

### 3.3. Measures

The interpretation and subsequent categorization of the interviews were guided by the study’s analytical framework, which categories are outlined in Table 3. This framework defines each dimension and provides guiding questions for analysis. Regarding *Institutional Logic (professional and market)*, both managers and employees of accredited hospitals were interviewed to identify which institutional logic is most present in hospital accreditation, assuming that they could potentially compete in the adoption process [27].

Regarding the *Adoption Objectives (legitimacy and efficiency)*, the focus was primarily on hospital managers, as they make strategic decisions on behalf of the hospitals. However, input from other interviewees was also considered to capture diverse perspectives.

For *Strategic Responses to Adoption (Conformity, Non-Conformity, and Customization)*, we sought to understand how hospitals responded to the accreditation process in practice. Despite all hospitals achieving accreditation, the extent of adoption and internalization varied.

Lastly, regarding the *Nature of Demands (Origins in Means or Goals)*, the goal was to identify conflicts or actions contrary to accreditation adoption within organizations. Emphasis was placed on understanding which aspects were prioritized by different participants.

**Table 3:** Analytical categories, dimensions, and questions used in the questionnaires.

Categories	Definitions of Dimensions for Each Category	Questions
Institutional Logics [2,12,23,37]	Professional Logic: Emphasizes healthcare professionals’ performance, advocating the quality of services, patient, and professional safety, and ensuring reputation and protection. Risks to this logic include compromised care quality and potential errors.	(a) Do you support implementing accreditation in the hospital? What improvements could accreditation bring to the hospital? (b) Can you identify the main challenges encountered during the accreditation process? (c) When choosing to join the Hospital Accreditation program, were there differences of opinions or any conflict among management members? (d) What were the main obstacles/problems that the hospital faced by the hospital during the adoption process?
	Market Logic: Focuses on the organization’s financial performance and market status. Key objectives include profit generation and organizational growth. The main threatening mechanisms are poor financial results, decreased performance, operational efficiency, and increased costs.	
Objective of Adoption [19,48]	Legitimacy: Aims to gain greater acceptance, respect, and status by stakeholders, including customers, suppliers, and competitors.	(a) When did the institution decide to pursue Hospital Accreditation? (b) How would you define Hospital Accreditation? What does it mean to you? (c) What factors influenced the decision to pursue Hospital Accreditation? Were there alternative options? (d) Why do you consider Hospital Accreditation important to the institution? (e) What did the institution hope to achieve by adopting Hospital Accreditation?
	Efficiency: Concentrates on enhancing the technical aspects of its operations, such as reducing costs, improving procedures, and increasing productivity.	
Strategic Responses [19,20,21,22]	Conformity: Involves full adherence to the Hospital Accreditation Program, maintaining compliance with the precepts and protocols required even post-certification.	(a) Were all requirements strictly met, or could they have been met through alternative methods? (b) After obtaining certification, were all processes maintained? (c) When the hospital is reassessed, what challenges arise? (d) Do you believe everyone in your department meets all the standards required by accreditation daily? (e) If you were visited by a certification agency auditor today, do you think the hospital would be accredited again?
	Non-conformity: The hospital predominantly rejects the practices established by the Hospital Accreditation program, retaining only those already practiced or having less impact than competing logics.	
	Customization: Primarily adopts the practices established by the Hospital Accreditation program but modifies them to suit the hospital’s actual needs.	
Nature of Demands [14,23,33]	Origin in the Means: Conflicts arise during the accreditation process, mainly in its implementation and organizational reproduction.	(a) What were the main obstacles/problems that the hospital faced during the adoption process? (b) Why did the institution decide to pursue Hospital Accreditation? Were there alternative options? (c) Who made the decision?
	Origin in Goals: Actions contrary to the decision to adopt accreditation; obstacles arise during the decision-making phase before implementation.	

Note: Five management-related questions were excluded from the respondents.

### 3.4. Categorization of Interview Excerpts

The in-depth interviews and observations were segmented and interpreted by both researchers. Categories were assigned based on the content analysis of the excerpts [59], aiming to identify similarities across narratives and categories [24,52]. Following established methodologies [60] used in previous studies, we ensured greater reliability in interpreting the segments by assessing the consistency of judgments between researchers using the Krippendorff alpha index, which yielded a high similarity score of 0.92. Any identified inconsistencies were resolved through double-checking by the authors [61]. Notably, the excerpts were categorized throughout interview texts, not solely limited to the responses related to each category.

Using the definitions in Table 3, each text segment was analyzed considering the analytical categories and their respective dimensions. With the help of a data spreadsheet, the segments were then highlighted, organized into rows in the same column, and categorized into four variables related to the broader categories and dimensions of the study. Exemplary excerpts were identified and extracted from the interviews to illustrate the authors’ assertions. To make the segments intelligible, structural changes were made in the translation from Portuguese to English to ensure clarity while preserving the original meaning. When, for example, the interviewee says that “*not all accreditation determinations were made*”, the excerpt was categorized as a customization in the accreditation process. Similarly, instances where physicians resisted implementing accreditation protocols highlighted conflict in the means category.

### 3.5. Analytical Strategy

Seeking enhanced study validity through methodological [62,63], we compared researchers’ interpretations of each text segment with the interplay of categories identified through multiple correspondence analysis (MCA). MCA serves as a formal method for analyzing the proximity and divergence of analytical categories, considering their recurrence patterns [64]. Its interpretation is facilitated by visually mapping the relationships between nominal categories.

To this end, we conducted multiple correspondence analyses using the SPSS version 25 statistical package. The visual representation was further refined using the Power BI application to enhance readability. In operational terms, all analytical categories were included as variables (Table 3), and a category map was generated from the scores on a two-dimensional place (see Appendix A. Analytical Category Scores). The MCA exhibited satisfactory adjustment, with dimension 1 yielding a Cronbach’s alpha of 0.739 (Inertia of 0.560) and dimension 2 of 0.645 (Inertia of 0.484), with all included categories displaying significant discrimination (*p* < 0.05).

It is imperative to note that the selection of MCA as a formal method stems from the necessity to examine the relationship between individuals and categories, conceiving them as mutually constituted within fields of action [65] or institutional frameworks [25]. This suggests that comprehending the actions, understandings, and justifications of organizational and professional actors in their context is broadened by going beyond verbal statements, as what is not said can also unveil how the hospital accreditation process is organized.

In this regard, we emulate Friedland et al.’s [25] approach by using MCA to elucidate how professional and market logics materialized amidst conflicts in accreditation, whether in processes or goals and how these aspects correlated with adoption objectives and responses in terms of conformity or non-conformity. So, the greater the proximity between the dimensions of the analytical categories, the more significant the overlap between them. Thus, greater distance indicates greater disparity between these categories.

To facilitate comprehension of the findings, we provide a synthesis of the MCA results, followed by descriptions of excerpts from interviews and researchers’ interpretations regarding the manifestation of analytical categories in the case studies. Finally, after interpreting the results, we aim for broader transferability [66], as well as more significant potential for analytical generalization [67] by formulating empirically verifiable propositions [68] to support new studies on hospital accreditation from the perspective of institutional logics (Section 5).

## 4. Results

Upon analyzing interviews with the 16 survey respondents, including managers and employees from four different hospitals, 187 text segments of texts were identified, representing the categories central to this research. Initially, we illustrate their relationships derived from the MDS, followed by the highlighting of pertinent excerpts.

### 4.1. The Convergence of Logics, Nature of Demands, Objectives and Strategic Responses

**Figure 1:**
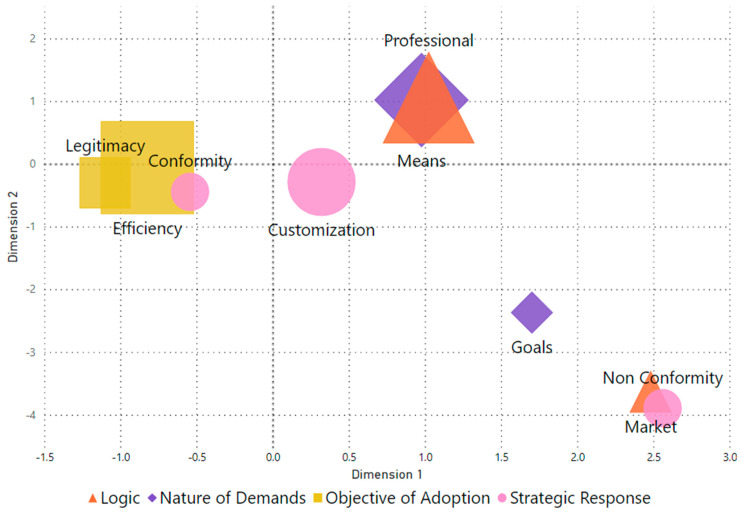
Multiple Correspondence Analysis of the researched categories. Note: Each geometric shape represents an analytical category, distinguished by different colors. The dimensions of the categories are depicted by various shapes on the two-dimensional graph, their sizes indicating the frequency of mentions. Dimension 1 is primarily influenced by adoption objectives, whereas dimension 2 is more affected by strategic responses. Both institutional logic and the nature of demands are prominently manifested in both dimensions.

To elucidate aspects pertaining to the expression of logic and other analytical categories extending beyond mere narrative analysis, we present the results of the multiple correspondence analysis (MCA) in Figure 1. Notably, the MCA not only validates the alignment observed among certain categories in the respondents’ statements but also highlights those exhibiting divergence.

For example, when respondents discuss the significance of adhering to hospital accreditation, they allude to the dual aim of enhancing efficiency and bolstering the hospital’s legitimacy. When addressing the necessity for adjusting accredited practices (Conformity), the reasons provided often revolve around professional mandates that pose challenges and necessitate tailored solutions. Furthermore, the convergence of arguments regarding adequacy due to professional requisites is more prevalent concerning conflicts arising from how things are done rather than with disagreements over their reasons.

In the bottom-right quadrant of the figure, arguments concerning non-compliance with the hospital accreditation process underscore economic considerations (Market Logic) as reasons for non-compliance. In turn, these arguments align more closely with conflicts over goals that appear inherently conflicting—for example, cost-cutting measures clashing against adherence to the accreditation’s detailed specifications. Therefore, the narratives surrounding each category are scrutinized to lend further depth to these synthesized findings.

### 4.2. The Expression of Professional and Market Logics in Accreditation

When examining the institutional logic category, insights from employees and certification agency auditors initially highlight the bureaucratic nature of the accreditation process, which entails a standardized procedure following precise protocols.

*“It is a certification that assesses whether the hospital meets the requirements [outlined] in the accreditation manual, with the aim of [ensuring] patient safety and organizational enhancement.”*

(CBA Auditor)

*“The auditor uses a checklist to evaluate the hospital based on the manual’s requirements. The score is determined by the degree of compliance for each assessed item, with a potential score [ranging from] zero, five, or ten.”*

(Hospital manager C)

Managers and employees were queried about the primary hindrances in the accreditation process and actions or facts that impeded or halted progress. Out of the 56 highlighted excerpts explaining the logic prevalent in the accreditation process, 91% are associated with professional logic, while only 9% are related to market logic. Owing to the discrepancies in the understanding of accreditation presented by these logics, challenges emerge, including resistance from physicians, nurses, and staff, as well as difficulties in team cohesion.

The research suggests that healthcare professionals, particularly physicians and nurses, wield significant influence over organizational practices and actions. Managers also underscored the necessity for all processes to be thoroughly negotiated with the medical and nursing staff. Otherwise, the accreditation process would not proceed as desired by the executive body.

*“The biggest difficulty is the commitment of the medical staff. They say they don’t need to improve because they’re already very skilled. It’s hard for physicians to recognize that they need to improve. Even scientific evidence shows that physicians are the last to comply with certification requirements. For this reason, when the certification process starts, the hospital immediately begins the work of convincing the medical staff.”*

(Hospital manager B)

*“The biggest challenge was aligning the activities required for certification with all the professionals in a harmonious way. Because we nurses depended on other professionals to complete our activities, it hindered the certification process.”*

(Nurse 1 from hospital C)

It is also noteworthy that, when discussing the professional ethics of individuals directly involved in patient care, the healthcare logic, which underlies professional logic, also manifests in the discourse of managers. This illustrates the prominence of professional logic in the accreditation process, even among managers.

*“The patient is the most important person in this hospital. I work for the patient. Everything I do is for the patient. So we had to adapt some of the things stated in the manual with the patient in mind.”*

(Hospital manager A)

The research also revealed that conflicts associated with market logic are primarily linked to financial investments, including expenditures on renovations, structural adjustments, and recruitment. Conflicts arising in board decisions regarding substantial investments were cited as major obstacles to attaining accreditation.

*“In 2001, there was a very stormy period in the hospital management, which led to the dismissal of many people. As a result, the accreditation process stalled for months.”*

(Hospital manager C)

*“At a certain point, changes had to be made in my sector to meet the certification requirements. However, the significant expenses required for these changes were not immediately authorized, resulting in a delay of the accreditation process until the hospital met the necessary criteria.”*

(Nurse 2 at Hospital C)

### 4.3. Balancing Legitimacy and Efficiency in Hospital Accreditation

They highlighted a pursuit for efficiency by hospitals in their decision to adopt hospital accreditation. Among the 35 excerpts collected in this category, approximately 65% solely emphasize efficiency as the primary objective of adoption. In the words of respondents, especially managers, the sought-after efficiency is linked to enhancing patient care quality, ensuring increased safety, and optimizing organization management.

*“The hospital aimed to enhance both the performance of its professionals and its operational processes. Its interest was adopting a certification model that is widely recognized as effective in its purpose of providing quality and safety in healthcare delivery.”*

(Hospital Manager B)

However, it became apparent that the adoption objective also reflects a desire to establish legitimacy within the hospital community. In particular, the research indicated that the sought-after legitimacy is more closely associated with garnering acceptance within the hospital organizational realm. Consistent with previous research [18,35,44], the interviews showed that accredited hospitals are keen on signaling the quality of their procedures, including physicians and nurses, as they serve as validators of hospital operations [69,70].

*“The hospital has always been committed to providing [high-]quality medical care that exceeds patient expectations.”*

(Hospital manager B)

*“It is a voluntary assessment process in which a healthcare organization, such as a hospital, agrees to submit its administrative and care processes to certification standards. If the hospital achieves the required compliance, it earns a seal, which means it has [established] a patient quality and safety program that sets it apart positively from its counterparts.”*

(Hospital manager B)

Additionally, the managers underscored their concern regarding attaining hospital accreditation even though patients may struggle to discern its direct impact on quality [6]. According to managers, most patients do not correlate the hospital’s accreditation status with its quality of care. Nonetheless, managers advocate for the accreditation process.

*“The patient sees the certification procedures but does not grasp their importance. I believe there is a lack of information […] Many [patients] find it strange to wear wristbands and may even complain about it. However, the clients don’t know that it is part of an accreditation protocol that increases the safety and care quality.”*

(Hospital manager A)

### 4.4. Conformity, Customization, and Non-Compliance in Hospital Accreditation

We also aimed to analyze how hospitals responded to accreditation requirements, especially concerning the integration of new methodologies, practices, and work routines. Interview reports indicate that 75% of the segments primarily highlighted a process of customization in accreditation. In other words, hospitals did not strictly adhere to all the protocols mandated by the accreditor, as already noted in the literature [36], especially in cases of resource scarcity [4] and when professional autonomy in procedure execution is maintained [10]. However, most of them were retained, while others were readjusted.

*“[…] in my routine, I had no trouble [implementing] many changes, especially because I was admitted while the accreditation was being implemented.”*

(Physiotherapist at hospital B)

Customizing hospital accreditation certification poses significant challenges during the revalidation process, which occurs every three years. The following excerpts show how hospitals had trouble adhering to all regulations and how there was a gradual relaxation over time, resulting in non-uniform adoption of protocols.

*“[…] you can’t fake all the protocols at all. You need to have an organized process. Now… it’s logical that, after the assessments are completed, you relax… After all, you can’t monitor everyone all the time, so it’s very difficult for you to maintain all the protocols. People tend to customize, so you have to keep working.”*

(Hospital manager A)

*“It is very difficult to get the accreditation certificate. But it is ten times harder to maintain it. We lose and regain processes all the time. We have to be sensitive and aware [to discern] when we lose the process. When we lose, everybody loses, it happens. You have to see if you are missing an important process that will affect something significant. It’s difficult to maintain it. It is hard to attain it, but that’s not the hardest part.”*

(Hospital manager D)

Instances showing some evidence of non-conformity, albeit rare (only three segments emerged), most pertained to a delay in accreditation activities rather than outright refusal to adhere to the accreditor’s directives. Nevertheless, the statements indicate a potential risk of non-conformity when financial interests are at stake.

*“[…] There was a period when they had to make changes in my department that required a substantial financial investment, which was not immediately approved, so the accreditation process was halted for a while until the hospital fulfilled the requirements.”*

(Nurse 1 Hospital A)

### 4.5. Conflicts over Means and Goals in Hospital Accreditation

In 8% of the text segments, conflicts related to goal-oriented demands were highlighted, often tied to the decisions regarding the continuation or cessation of the accreditation process, as well as conflict between hospital management and executives responsible for leading accreditation goals.

*“During the accreditation process, there were changes in leadership. Until the new management understood and ratified the process […], there were several elections along the way […] changes in nursing leadership, changes in nursing directors […] Then, there was a discontinuity, a disrupture in the process.”*

(Hospital manager C)

The narratives also underscored that managers’ primary concern lay in addressing the demands of stakeholders, aiming to avoid threatening hospital operations, especially when they involved government entities wielding influence, even if indirectly.

*“Shortly after José Serra became the Minister of Health, he mandated that we obtain certification from ONA. Despite our belief that the CBA is superior to ONA for several reasons, we underwent evaluation by ONA under the Ministry’s directive. After the change in government, we no longer required ONA and reverted to JCI (CBA). Later, we scheduled a visit and obtained accreditation through CBA.”*

(Hospital manager C)

As observed in all hospitals, there was a notable trend toward segmenting activities related to the adoption process [27]. These activities, often encompassing issues of financial efficiency and operational concerns, were not always communicated effectively with the professional body. Thus, while professionals were tasked with executing intermediary activities, irrespective of their agreement, managers compartmentalized their concerns primarily in terms of goals, encompassing both efficiency and legitimacy [17]. This is exemplified in the response of a hospital manager when questioned by the researcher regarding the pressure for accreditation.

*“[Researcher’s question] Did the hospital’s certification by ONA owe to external pressure? [Manager D’s answer] Yes. Otherwise, we wouldn’t have changed. With the change in management and, consequently, the change in certification, there were days when 29 auditors were working at the same time. We couldn’t work. It was a very troubled period.”*

(Hospital manager D)

Consistent with prior research [18,71], the majority of segments detailing demands during the accreditation program implementation process focused on means and procedures (92% of mentions). One such challenge encountered is exemplified in the following account.

*“The accreditation manual was updated last year, so we anticipate that the accreditation process will be less challenging next year. However, some workers have abandoned certain procedures. When you are close to achieving certification, you may need to catch up on these procedures to successfully obtain it. Therefore, the process is slow and requires a systematic approach. It demands organization […].”*

(Hospital manager A)

Despite encountering numerous challenges from both professional and management teams, organizations tend not to persevere with the accreditation process, even when it extends beyond anticipated timelines.

*“For instance, I saw a hospital that had been ISO-certified for eight years. When they kicked off the accreditation process, they already had well-structured documentation. But it still took them almost three years to achieve accreditation. Since there is so much more detail about patient care but not necessarily about documentation, I saw [the case of] a hospital that took nine years. But with great difficulty [anyway].”*

(ONA Auditor)

*“The hospitals I worked at achieved accreditation. Three private hospitals where I started working from the first day. One took two and a half years, another three and a half years, and the third three years. All three attained accreditation certification. In this one public hospital, I worked for seven years on the accreditation process. After I left, it took another two years to complete the accreditation […].”*

(CBA Auditor)

The accounts also underscore the pivotal role of medical professionals and nurses, who, despite their subordination to managers and executives, do not readily comply with the demands of the governing body to facilitate the accreditation process, nor do they compromise their professional identity.

*“Over time, of course, you will say that there was no resistance… Of course, there was some resistance, but [it was] because it was the will of management, and we said it was something important, people understood. So, the decoding [the idea] that this [process] is going to improve the quality of patient care is important. So, obviously, when we are visited today, there is no stress… But it was stressful. My God, how is this or that process? That no longer exists because it has already become part of everyday life.”*

(Hospital manager A)

*“The accreditation certificate is sometimes seen as a bargain. If the worker does what is required, then he wants something else. They don’t have to want anything! When we explained to them that what the protocol requires is what we must do.”*

(Hospital manager A)

According to a physiotherapist at Hospital B, physicians showed even greater resistance: “*The main difficulty encountered was the more active participation of* physicians *who were always very critical*”. This was primarily due to the fact that accreditation entails increased utilization of control and bureaucratization systems. This not only challenges the autonomy of the medical professionals but also demands skills that they often lack awareness of. As one of the physicians (Hospital B) remarked: “*It was mainly due to increasing bureaucracy […] Bureaucracy increased the number of reports and other similar actions*”. Furthermore, there was a consensus that the greatest challenge of accreditation was fostering teamwork, not the division of work itself, but more fluid and collaborative coordination, aligning with findings from other studies [2,18].

## 5. Developing Empirical Propositions on Logic Conflicts and Demand Nature in Hospital Accreditation

The analysis of narratives and correspondence between categories enabled the development of empirical propositions that can inform future studies aiming to validate these results [68]. These prepositions also highlight gaps in understanding how market and professional logic manifest in hospital accreditation processes.

Firstly, the results indicate that when professional logics predominate in the accreditation process, hospitals may customize the actions required by the accrediting organization. As some resistance from professionals was anticipated [23], the interviews illustrated that the prominence of professional logic leads to resistance from clinical staff, a lack of leadership, and a lack of coordination between teams. Consistent with the literature, customization arises because professionals need to reinterpret how work is executed [13], necessitating hospitals to collaborate with the medical staff and nurses to ensure that new practices are authentically embraced in a manner that validates their professional identity [16].

*“Sometimes we have to include bosses in these groups if they are not contributing effectively.”*

(Hospital manager C)

*“The mobility of professionals has made it difficult, not just for physicians. In the administration, there is often a sentiment of resistance towards these professionals [physicians].”*

(Hospital manager D)

There are reports indicating that the hospital has adopted the standards and protocols mandated by the accrediting organization. However, some of these practices are lost during the course of the process and its replication. Both managers and certification agency auditors noted that constant monitoring by team leaders and support from management stand out as a means of seeking the institutionalization of practices included by hospital accreditation. Nonetheless, research has revealed that achieving full conformity is complex and time-consuming. Expressions such as “most”, “almost always”, and “practically” were used by interviewees to illustrate that the adoption of accreditation did not result in complete conformity.

Another significant factor in customizing the accreditation process based on professional logic is the segmentation among groups of professionals within hospital organizations [27]. The high level of technical expertise among professionals with different specialties leads to small “fiefdoms” within the organization, complicating the interaction between teams, which is consistent with previous studies [23,33]. Without integration among professionals, monitoring actions and compliance with the practice required by the hospital accreditation program becomes more challenging.

In this regard, both case analyses and the existing literature point to two reasons for the hybridization of logics that result in customization [23,27]. Firstly, doctors, nurses, and other health professionals are acquainted with the accreditation process, but they disagree on the methods by which activities should be conducted, leading to work compartmentalization [23]. Secondly, the only viable option for compartmentalizing work is to segment it [27] into interconnected parts within the hospital organization, as medical care is inseparable. Given these relationships, we propose the following:

**Proposition** **1a:** *In hospital accreditation processes, the prominence of professional logic increases the likelihood of customizing certified practices*.

**Proposition** **1b:** *In the presence of distinct professional logics, there is an increased probability of segmenting certified practices*.

Secondly, when hospitals address market demands involving investment decisions and resource mobilization, there tends to be a pause in adapting practices to accreditation standards, causing the organization to deviate from the accreditation protocol during this period.

*“We only stopped the process when we had to put off the auditors’ visit to evaluate the protocols, for in the last week, we had to make many adjustments that required a huge expense. This took a while to be approved.”*

(Hospital manager D)

*“Obtaining the hospital accreditation certificate is not cheap. It’s not cheap because it involves money that must be paid for the methodology. It involves paying for international certification agency auditors. When we have a problem with the budget, this creates an interruption in the process.”*

(Hospital manager C)

The reports led us to conclude that the likelihood of non-conformity is more significant when there are conflicts related to market logic. This is because aspects related to this logic proved to be more challenging to overcome throughout the organization. After all, it always involves a management position that prioritizes financial matters, such as investments in works, structural readjustments, and costs, to the detriment of other aspects of accreditation [72]. Suppose the tension between the market and professional logic cannot be resolved. In that case, the dominant logic (typically the market logic) can block the hospital accreditation process [27], resulting in non-compliance. Consequently, we propose:

**Proposition** **2:** *In the presence of conflicts between market and professional logic, the greater the prominence of the market logic, the higher the likelihood of non-compliance with practices*.

Thirdly, when evaluating the nature of the demands [23] in the hospital accreditation process, the research indicated that the vast majority originate from the mean activities. The conflicting aspects between the accreditation process ‘ needs and professionals’ identity led hospitals to customize their certified practices, not fully adopting the protocols. However, they adopted enough to be approved, creating a hybrid type of organizational practice that blends [27] and bricolages [73] professional aspects with those of certification. This leads us to propose:

**Proposition** **3a:** *When the focus of demands is on means, there is a greater chance of customization in hospital accreditation processes*.

In such cases, professional logic tends to prevail, as the main conflict factors are linked to the practice of technical activities and work routines [23,33]. Furthermore, the customization caused by the strong presence of professional logic in the process shows that the hospital, when faced with obstacles during the certification process, remains on the path set in the initial objectives, even if this decision means consuming more time to attain accreditation. Therefore, we propose that:

**Proposition** **3b:** *The greater the centrality of professional logic, the greater the likelihood of conflicts occurring in the demands of mean activities*.

However, when conflicts focus on goals generally related to market logic [2], the organization tends not to conform to the process, usually pausing it or postponing adoption. Thus, when the conflict focuses on the goals and the purpose of adoption, the process tends to stall or be interrupted until the issue is resolved. So we propose:

**Proposition** **4a:** *In hospital accreditation processes, when the focus of demands is on goals, there is a greater chance of non-conformity*.

**Proposition** **4b:** *The greater the centrality of market logic, the greater the likelihood that conflicts will focus on goals*.

Finally, the analyses showed that the hospital accreditation process, even though voluntary, did not lead to conflicts related to the pursuit of legitimacy at the expense of efficiency despite such disputes being recurrent [18,19,37]. It turns out that when there is no pressure for mandatory certifications, organizations, including hospitals, do not have a compelling reason to adopt practices such as certification. This is primarily because they are also striving for improvements in efficiency and quality. Thus, the voluntary nature of hospital accreditations in Brazil does not create contradictions between the objectives of adoption [74,75]. If a regulatory body were to impose a centralized demand for hospital certification, conflicts in compliance would likely arise [75], leading to divergences between efficiency and legitimacy [23]. Therefore, we propose that:

**Proposition** **5a:** *When hospital accreditation processes are voluntary, conflicts between efficiency and legitimacy objectives are less likely*.

**Proposition** **5b.** *The greater the imposition of hospital accreditation, the greater the likelihood that adoption will be ceremonial and guided by legitimation interests*.

## 6. Discussion

### 6.1. Summary of Findings and Theoretical and Practical Implications

This study aimed to investigate how professional and market logic, along with conflicts between institutional demands, impact compliance with hospital accreditation. To achieve this goal, we conducted a qualitative multiple-case study involving four Brazilian hospitals to unravel the intricacies of this process. Through data collection and analysis, we observed that institutional logics not only significantly shape the strategic responses of organizations but also determines the type of strategic response—conformity, non-conformity, or customization –depending on which logic is directly at play.

The research also highlighted that despite the significant conflicts between institutional logic in the accreditation process, organizations tend to achieve substantive rather than symbolic results. This underscores a crucial theoretical contribution of our work. Considering institutional logic and its variations is essential when assessing organizational strategic responses. We found that when professional logic predominates, there is a greater likelihood of customization, whereas when market logic prevails, the organizations tend to deviate from accreditation practices.

Numerous studies have explored strategic responses within the organizational field. Oliver [21], for instance, delves into institutional factors such as causes, constituents, control, context, and content; Pache and Santos [23] examine variables such as internal representation and the nature of the demands. However, despite these and other works on strategic responses and institutional logic, research directly correlating these two categories of analysis remains scarce in organizational studies. This study has sought, in a way, to emphasize that institutional logic and its variations profoundly influence the strategic responses of organizations.

In the realm of hospitals, our research contributes to a better understanding of why organizations choose and implement accreditation to enhance quality in Brazilian healthcare. We identified tangible objectives tied to actions targeting both employees and patients. Regarding the legitimacy of the program, while patients may still have limited awareness of hospital accreditation or other quality programs in the field, and despite it not being a mandatory process, our findings reveal the normative nature of the practice, particularly among hospital organizations. The pursuit of efficiency also emerged as a crucial factor in our research, indicating that Hospital Accreditation transcends mere ceremonialism, striving to align certification objectives with those of hospital organizations.

The propositions delineated in this study furnish a robust framework for steering future research endeavors, fostering a deeper comprehension of the intricate dynamics within hospital accreditation processes. As highlighted, the voluntary nature of hospital accreditations in Brazil appears not to engender significant conflicts between efficiency and legitimacy. This implies that hospital organizations can pursue accreditation as an effective means of improving both efficiency and service quality without encountering substantial contradictions between these aims.

From a practical standpoint, the implications are equally consequential. Hospital managers stand to derive substantial benefits from the propositions, gaining insights into how market and professional logic influence the accreditation process. In alignment with studies such as Reay et al. [18] and Rossoni et al. [37], hospital accreditation frequently entails striking a balance between efficiency and legitimacy. Managers can employ these propositions as navigational aids for reconciling these competing demands.

For example, in scenarios where professional logic is prominent, managers should anticipate resistance from healthcare professionals and a lack of leadership. Resistance from professionals is a recurrent challenge in hospital accreditation contexts, a point reiterated consistently in the literature [23]. To foster compliance, managers can implement strategies that involve clinical staff in reviewing and adapting practices, emphasizing the significance of validating professional identity.

Similarly, when confronted with demands rooted in market logic, managers should be aware of the tendency to halt the adaptation process owing to financial constraints. Financial considerations often pose formidable obstacles in the adoption of practices, including hospital accreditation [19,72]. This can encourage the search for financing and investment strategies that facilitate the accreditation process while aligning the organization’s financial objectives.

The practical implications also encompass grasping the nature of the demands inherent in hospital accreditation. Managers can embrace more tailored approaches by recognizing that the nature of demands can impact the level of customization and conflicts. In line with the observations of Skelcher and Smith [27], multidisciplinary teams within hospitals often grapple with integration challenges. When the demands primarily focus on means, teams can concentrate on adapting technical practices and work routines. Conversely, when conflicts center on goals, managers must address these issues proactively before advancing with the accreditation process, thereby averting significant interruptions.

In the context of hospital accreditation compliance, the interplay between professional and market logic with institutional demands presents a SWOT analysis with the following key points: Strengths encompass a robust emphasis on high-quality patient care and medical expertise. Weaknesses entail hurdles in cost containment and flexibility stemming from the rigidity of professional standards. Hospitals stand to gain from embracing technological innovations to enhance efficiency and forging alliances to pool resources. However, they also confront economic pressures to curtail costs and intense competition that may prioritize market-driven healthcare practices over professional guidelines.

### 6.2. Limitations and Suggestions for Future Studies

Despite making significant strides in understanding the intricacies of organizational compliance and hospital accreditation, we acknowledge some limitations that merit consideration when interpreting the results. Firstly, our study focused on hospitals situated within a specific context, potentially constraining the generalizability of findings to other regions or healthcare systems characterized by different standards [26]. Also, the applicability of the results may be limited to hospitals in different countries or healthcare systems, necessitating comparative studies across varied contexts to assess the external validity of the propositions developed.

Secondly, the research approach employed in this study was qualitative, centering on multiple case studies conducted in Brazilian hospitals. While this approach yields in-depth insights, it may curtail the generalizability of the findings to settings or contexts beyond the specific hospitals under study.

Thirdly, we recommend delving into the influence of other key actors, such as patients and their families, on the dynamics of hospital accreditation [27]. Their perspectives and expectations can wield significant influence on organizational compliance. Furthermore, additional investigations could explore the strategies adopted by hospital managers to navigate the complex interplay between different logics, demands, and objectives [26]. Understanding how managers can promote alignment between different logics and mitigate conflicts is crucial for enhancing the efficacy of the hospital accreditation process.

Lastly, future studies could explore the evolution of accreditation and compliance practices over time, considering how contextual factors and changes in legislation can affect the dynamics of hospital organizations [27]. Adopting a long-term perspective would furnish a more holistic understanding of the intricate relationships between logic, demands, and objectives in hospital accreditation.

## 7. Conclusions

The research sheds light on how the identification of actors with specific logics influences the prioritization of demands of a particular nature, corroborating previous findings [23]. Furthermore, within the multifaceted and sometimes conflicting landscape of various logics in hospital organizations, it is remarkable how these logics tend to compartmentalize to mitigate extensive conflicts [26,27]. When professional logic takes precedence, conflicts emerge concerning means, resulting in the customization of accreditation practices. In contrast, in scenarios dominated by market logic, conflicts center around goals, leading to non-conformity. These findings deepen our understanding of how logic shapes the attention given to specific demands and, more significantly, how they impact compliance with hospital accreditation standards, thereby enriching the body of knowledge in the field.

## Figures and Tables

**Table 1:** Ideal types of institutional logic relevant to the hospital accreditation process.

Logic	Market	Professional
Guidance	Focuses on business strategies, market competition, and return on investment.	Based on norms and standards established by professional health associations and bodies.
Main Emphasis	Maximizing operational efficiency and financial profitability.	Providing high-quality healthcare based on best practices and professional standards.
Motivation	Obtaining profit, optimizing resources, and expanding the market.	Commitment to professional ethics, continuous improvement in the quality of care, and patient well-being.
Threats	Increased costs and loss of customers.	Reduction in the quality of services and professional malpractice.
Basis of authority	Owners, investors, and managers.	Professionals with the most outstanding technical and scientific reputations, especially physicians and nurses.
Source of Legitimacy	Position of the hospital in the market compared to other competitors.	Status of the hospital in the professional community, available resources, and structure quality.
Performance Measurement	Based on financial indicators such as profit margins, revenue, and market share.	Based on quality indicators, patient satisfaction, and adherence to professional standards.
Decision Making	Considerations regarding efficiency, competition, and financial return often guide strategic decisions.	Physicians and healthcare professionals frequently make clinical and operational decisions to meet patient needs best.
Organizational structure	Organizational structure is often oriented toward management, finance, and operations functions.	Organizations are often structured around medical specialties and multidisciplinary teams.
Relationship with Patients	Attention to customer satisfaction, attracting and retaining patients, and maximizing the customer life cycle.	Focus on providing high-quality care and building trusting relationships with patients.
Regulation	Subject to government regulations, including compliance with health laws and healthcare regulations.	Subject to professional regulations, standards of practice, and healthcare licensing bodies.
Underlying Logic	Managerial	Healthcare

Note: Prepared by the authors, based on Andersson and Liff [13], Anthony et al. [1], Conceição et al. [2], Pouthier et al. [17], and Thornton et al. [12].

**Table 2:** Research participants.

Hospital, Nature, Year of Foundation and Accreditor	Interviewed	Number of Interviews	Total Recording Time
A (Private), 1988, ONA	Manager A	4	236 min
	Physician A	2	54 min
	Nurse 1 A	2	55 min
	Nurse 2 A	3	41 min
B (Private), 1930, CBA	Manager B	4	190 min
	Physician B	1	25 min
	Physiotherapist B	2	74 min
	Nurse B	1	22 min
C (Public), 1994, ONA	Manager C	5	274 min
	Physician C	2	49 min
	Nurse 1 C	1	81 min
	Nurse 2 C	3	171 min
D (Public), 1900, CBA	Manager D	4	212 min
	Physician 1 D	1	12 min
	Physician 2 D	2	44 min
	Nurse 2 C	2	87 min

Note: All interviews were recorded and transcribed, maintaining the anonymity of the respondents.

## Data Availability

The data used during this study are available from the corresponding author, upon request by email.

## Appendix A. Analytical Category Scores (MCA)

**Variable**	**Category**	**Dimension 1**	**Dimension 2**	**Frequency**
Adoption Objective	Efficiency	−0.8257674762	−0.0533433566	28
Adoption Objective	Legitimacy	−1.103079125	−0.294654722	7
Strategic Response	Conformity	−0.544897165	−0.438805406	3
Strategic Response	Customization	0.318283225	−0.281455581	14
Strategic Response	Non-Conformity	2.557873692	−3.887091214	3
Institutional Logic	Market	2.479505625	−3.627207731	4
Institutional Logic	Professional	1.02216587	1.063648106	27
Nature of Demands	Means	0.974744716	1.024762878	29
Nature of Demands	Goals	1.699802394	−2.365275414	4
